# Efficacy of iron-silver bimetallic nanoparticles to enhance radiotherapy

**DOI:** 10.1007/s00210-023-02556-9

**Published:** 2023-06-08

**Authors:** Marwa M. Afifi, Reem H. El-Gebaly, Ibrahim Y. Abdelrahman, Monira M. Rageh

**Affiliations:** 1 Biophysics Department, Faculty of Science, Cairo University, Cairo, Egypt; 2 Egyptian Atomic Energy Authority, National Center for Radiation Research and Technology, Cairo, Egypt

**Keywords:** Radiation therapy, Nanoparticles, Radiosensitizers, Ehrlich tumor, Oxidative stress, DNA damage

## Abstract

Radiotherapy (RT) is one of the primary cancer treatment methods. Radiosensitizers are used to enhance RT and protect healthy tissue. Heavy metals have been studied as radiosensitizers. Thus, iron oxide and iron oxide/silver nanoparticles have been the main subjects of this investigation. A simple honey-based synthesis of iron (IONPs) and iron-silver bimetallic nanoparticles (IO@AgNPs) were prepared followed by characterization with transmission electron microscope (TEM), absorption spectra, vibrating sample magnetometer (VSM), and X-ray diffraction (XRD). Additionally, Ehrlich carcinoma was induced in 30 adult BALB/c mice and divided into 6 groups. Mice of group G1 were not treated with nanoparticles or exposed to irradiation (control group), and group G2 and G3 were treated with IONPs and IO@AgNPs respectively. Mice of group G4 were exposed to a high dose of gamma radiation (HRD) (12 Gy). Groups G5 and G6 were treated with IONPs and IO@AgNPs followed by exposure to a low dose of gamma radiation (LRD) (6 Gy) respectively. The impact of NP on the treatment protocol was evaluated by checking tumor growth, DNA damage, and level of oxidative stress in addition to investigating tumor histopathology. Additional research on the toxicity of this protocol was also evaluated by looking at the liver’s cytotoxicity. When compared to HRD therapy, combination therapy (bimetallic NPs and LRD) significantly increased DNA damage by about 75% while having a stronger efficacy in slowing Ehrlich tumor growth (at the end of treatment protocol) by about 45%. Regarding the biosafety concern, mice treated with combination therapy showed lower alanine aminotransferase (ALT) levels in their liver tissues by about half the value of HRD. IO@AgNPs enhanced the therapeutic effect of low-dose radiation and increased the efficacy of treating Ehrlich tumors with the least amount of harm to normal tissues as compared to high radiation dosage therapy.

## Introduction

Radiation therapy is still a popular cancer treatment option, along with surgery and oral medications. More than 60% of patients with malignant tumors receive radiation therapy, and only 40% of these treatments are successful (Wang et al. 2018a, b). However, several adverse effects have been noted. Regarding radiation physics, recent advancements in radiation delivery methods, such as intensity-modulated radiotherapy and image-guided radiotherapy, to reduce the toxicity of normal tissue and increase local control rate through safe dose-escalation, various side effects, and an adequate therapeutic effect cannot be achieved due to several reasons, such as the size of the tumor is small, located in the brainstem, or adjacent to critical structures (Liu et al. 2018a, b). To overcome these problems, radiosensitizers are used to enhance the therapeutic advantages of low doses of radiation and raise the tumor’s radiation sensitivity (Wang et al. 2018a, b). Metal-based nanoparticles have been suggested as a novel way to raise the therapeutic index of radiation therapy by increasing the generation of free radicals and reactive oxygen species (ROS) (Liu et al. 2016). ROS can damage DNA and lipids, as well as disrupt signal transduction, resulting in cell death if the damage is severe enough that it cannot be repaired (Hauser et al. 2016). The response of metal nanoparticles to ionizing radiation is not the same as most organic polymers used in chemotherapy as the chemical bonds of such polymers are damaged by radiation while most nanomaterials accept or discharge photons and electrons without undergoing significant change in their basic structure (Paunesku et al. 2015). Among the transitory metal oxides, iron oxide nanoparticles or magnetite (IONPs) have higher cytotoxicity and strongest magnetic properties and because of their good biodegradability and biocompatibility features, their production and fabrication have long been a research goal as a radiation sensitizer in a variety of cancer cell lines (Liu et al. 2018a, b, Najafpoor et al. 2020, Rashid et al. 2019 and Klein et al. 2012). Klein et al. (2014) reported that IONPs could be employed as radiosensitizers after treating the MCF-7 cells by 3 Gy, which increases the ROS.

Also, silver nanoparticles are well known for their different applications, especially their anticancer effects. The primary mechanism of their toxic effects is mediated by the ions released from silver nanoparticles which cause the overproduction of free radicals that lead to oxidative stress (Rageh et al. 2018). In addition to their cytotoxicity, they have been getting to be another investigation hotspot within the field of radiation as they serve as radiosensitizers and enhancers for radiotherapy (Zhao et al. 2019).

The combination of IONPs and AgNPs in one hybrid nanostructure (IO@AgNPs) introduces a promising methodology for biomedical applications. It allows the distinct properties of each nanoparticle component to be combined and improved their individual features, resulting in multifunctional nanoparticles (Pieretti et al. 2020). For example, the research done by Zhang et al. (2018) indicates a high-performance radiosensitizer of iron-silver bimetallic nanoparticles.

Biosynthesis nanoparticles using natural resources as reducing and capping agents have been considered more environmentally friendly and cost-effective instead of production that uses toxic chemical methods due to their distinct optical properties and stability in an aqueous solution (Al-Asfar et al. 2018). Recently, natural honey has been used for nanoparticle preparation due to its ingredients that can function as antioxidants which play an impressive role in the treatment of cancer (Philip 2010).

There have been numerous in vitro studies with the utilization of nanoparticles for radio sensitization and there are few in vivo studies (Paunesku et al. 2015). Therefore, this work focuses on studying the efficacy of iron nanoparticles and the privileges of combining two metal nanoparticles (silver and iron), IO@AgNPs, as radio enhancers in vivo to treat solid Ehrlich carcinoma in Balb/C mice.

## Materials and methods

Silver nitrate (MW 169.87), iron (III) chloride hexahydrate (FeCl_3_·6H_2_O), iron (II) chloride tetrahydrate (FeCl_2_·4H_2_O), and sodium hydroxide NaOH (99.0%) were used without further purification and obtained from Sigma-Aldrich (Schnelldorf, Germany). Natural honey was obtained from an Egyptian honey apiary. Lipid peroxide (Malondialdehyde) CAT. no. MD2529 and total antioxidant capacity CAT. no. TA2513 were obtained from Biodiagnostic Company (Giza, Egypt).

### Preparation of IONPs and IO@AgNPs

Iron oxide nanoparticles (IONPs) were prepared accordingly to the method described in Rasouli et al. (2018) with further modification. Briefly, 1.6 g of iron III and 0.9 g of iron II were dissolved in 50 mL distilled water under vigorous stirring at room temperature (MS-20D magnetic stirrer, Dihan, Korea) for 1 min. A total of 5 mL of NaOH (26%) was titrated against the solution and left for 10 min under stirring; 5 g of honey (as a coprecipitating and stabilizing agent) is then added and the temperature was elevated to 50 °C under continuous stirring for 20 min. The resulting black suspension from iron nanoparticles was formed, separated using a permanent magnet, washed with deionized water, and finally dried in an oven at 60 °C.

Iron oxide/silver nanoparticle IO@AgNPs were synthesized according to the previously described method in Elbialy et al. (2014) with further modification. Briefly, 0.25 g of honey was added to a 40-mL boiled silver nitrate solution (1 mM) under a magnetic stirrer then 30 mL suspension of IONPs was added. After 10 min, the color started to change from colorless to green. The stirring continued for 20 min and after the suspension cooled to room temperature, IO@AgNPs were formed and separated by a permanent magnet.

### Characterization of IONPs and IO@AgNPs

The size and morphology of IONPs and IO@AgNPs were obtained by transmission electron microscope (TEM) (JEM 1230 electron microscope. Jeol, Tokyo, Japan). A drop of nanoparticles was connected to a carbon lattice coated with copper and the overabundance sample was drawn off with filter paper. The lattice was cleared out 5 min to dry at room temperature shortly before starting the examination.

The crystal structures of IONP and IO@AgNP nanoparticles were obtained through powder X-ray diffraction (XRD) (XRD model XPERT PRO-PANALYTICAL-Netherland).

The absorption spectra of IONPs and IO@AgNPs samples were measured using a spectrophotometer (Jenway 6405, Barloworld Scientific, Essex, UK).

The magnetic properties of IONP and IO@AgNP samples were measured by a vibrating sample magnetometer (VSM) (VSM, model LakeShore 7410).

### Animal groups and treatment protocol

Adult BALB/c mice of average weight 30 g and 8–10 weeks of age (obtained from the National Cancer Institute “NCI,” Cairo University) were injected subcutaneously in the right thigh with 250 µL cell suspension of Ehrlich tumor (total cell count of about 2–2.5 × 10^6^ cells/mL). The tumor was established in a single and solid form as described by Abdelrahman et al. (2020). Animal handling and care were performed according to the guidelines for the Care and Use of Laboratory Animals, Cairo University Institutional Animal Care and Use Committee (CU-IACUC), application number CU/I/F/15/19. Ten days after tumor cell injection, 30 mice were randomly divided into six groups (5 mice in each group). Subsequently, the treatment protocol was run every 3 days during this period (9 days) as follows:

**Table:** 

Mice groups	Treatment
G1	Mice were injected intratumorally (IT) with 200 μL saline
G2	Mice were injected IT with 200 μL IONPs (0.8 mg/mL)
G3	Mice were injected IT with 200 μL IO@AgNPs (1.6 mg/mL)
G4	Mice were irradiated with 12 Gy of X-rays fractionated equally into three sessions
G5	Mice were injected IT with 200 μL IONPs (0.8 mg/mL) pre 15 min irradiated with 6 Gy of X-rays fractionated equally into three sessions
G6	Mice were injected IT with 200 μL IO@AgNPs (1.6 mg/mL) pre 15 min irradiated with 6 Gy of X-rays fractionated equally into three sessions

### Radiation facilities

For irradiation, mice were placed in a well-ventilated container and subjected to whole-body irradiation using a Cs^137^ gamma source with a dose rate of 0.33 Gy/min. The source used was located at the National Center for Radiation Research and Technology (NCRRT) in Cairo, Egypt, and manufactured by Atomic Energy of Canada Limited, Ontario, Canada.

When the treatment was complete, the mice’s lives were terminated. Their tumor tissues and liver were swiftly taken out, washed with isotonic saline, divided into three sections, and utilized for assessment.

### Oxidative stress

In cold phosphate buffer, a portion of tumor tissues was homogenized from all mice groups. After centrifugation at 4000 rpm (VS18000 M; Vision Scientific, Korea), for 10 min, the supernatants were utilized to quantify malondialdehyde (MDA) and total antioxidant capacity (TAC) levels. The levels of MDA and TAC were evaluated using lipid peroxide (malondialdehyde) CAT. no. MD2529 and total antioxidant capacity CAT. no, TA2513 kits respectively according to the manufacturer’s instructions (Biodiagnostic Company, Giza, Egypt).

### Comet assay

Comet assay was utilized to evaluate the early stage of apoptosis and DNA damaging in cancer cells. Tumor tissues from all experimental groups were used for the determination of DNA damage by the method described by Rageh et al. (2018). A fluorescent microscope with a magnification power of 400 was used to investigate comets. The length of DNA migration and the percentage of DNA in tail and tail moment were measured in 50 cells using the Comet 5 image analysis software created by Kinetic Imaging, Ltd. (Liverpool, UK) coupled to a CCD camera.

### Tumor size

During the treatment protocol (9 days), tumor size was monitored for all the experimental groups. Tumor size was measured at day 0 when the pulp started to appear (about 200–300 mm^3^) using a digital caliper and the volume was calculated according to the equation described in Faustino-Rocha et al. (2013).

*V* = (*W*^2^ × *L*)/2, where *L* is the major tumor axis, *W* is the minor tumor axis, and *V* is the tumor volume. At the end of the treatment protocol, all the mice were anesthetized with Thiopental sodium (50 mg/kg) (Abdelrahman et al. 2020). Blood samples were collected and sacrificed; tumor tissues and liver were quickly removed from each group, washed with buffer saline, and divided into parts for different evaluations.

### Histopathological examination

Tumor tissues and liver organs from all treatment groups were fixed in 10% neutral buffered formalin, immersed in paraffin blocks, and sectioned and stained with hematoxylin and eosin (H&E). All tissue sections were observed using a light microscope (Olympus BX50, Japan) that was connected to a digital camera (Canon).

### Alanine aminotransferase (ALT) level analysis

Blood samples were collected from the heart vein of each mouse and transferred to a serum-separated tube, allowing the serum to clot and centrifuge to separate the serum. The levels of ALT in the blood were measured from the prepared serum samples.

### Statistical analysis

Data were collected from at least three separate studies and expressed as mean ± SD. To determine statistical significance (*P* < 0.05), a one-way analysis of variance (one-way ANOVA) and least-significant difference (LSD) test were performed using SPSS v.19.0 for Mac.

## Results and discussion

In most cancer treatment methods, RT is combined with chemotherapy. However, the size and location of the tumor restrict the radiation dose that is prescribed. Modulating radiation sensitivity and reducing side effects are issues that must be overcome to improve the therapeutic impact of tiny doses of radiation. Radiosensitizers are substances that, when combined with a low dose of radiation, enhance the killing effect of tumor cells by generating free radicals which can induce cellular stress and biomolecule injury. Radiosensitizers typically have less impact on healthy tissues. Several studies reported that radio sensitization effects are caused by metallic nanoparticles with a high atomic number. In addition, heavy metal-based nanosized radiosensitizers are highly effective where the size and shape of the nanoparticles are closely related to the intracellular uptake of nanoparticles, where small and spherical nanoparticles are more likely to enter the cells causing a higher toxic effect (Liu et al. 2018a, b and Rashid et al. 2019). Chaves et al. (2017) informed that the uptake of nanoparticles in breast cancer cells is more effective than in normal cells. Thus, the current study focused on the efficacy IONPs and IO@AgNPs as radiosensitizers through the modulation of Ehrlich carcinoma.

**Fig. 1:**
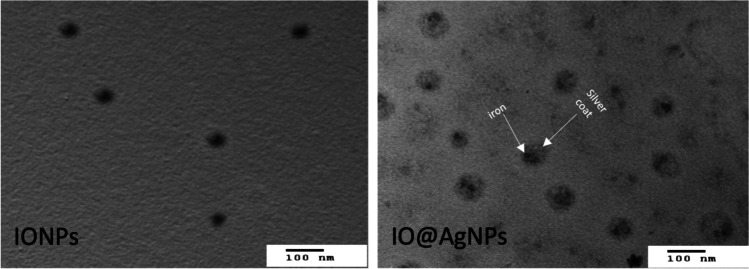
Transmission electron microscope (TEM) images of iron oxide nanoparticles (IONPs) and iron-silver bimetallic nanoparticles (IO@AgNPs)

TEM was used to visualize specimens and generate a highly magnified image to examine the morphologies of nanoparticles and measure the particle size. Figure 1 shows that the IONPs were spherical in shape with an average particle size of 12 ± 2 nm. The IO@AgNPs appear semi-spherical, well dispersed, and uniform in size with a shadow silver nanoparticle coating the dark iron nanoparticles with an average size of 40 ± 0.9 nm.

**Fig. 2:**
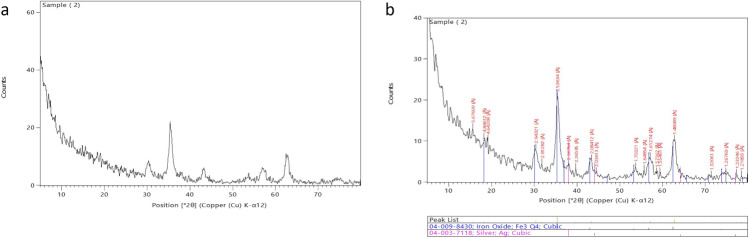
X-ray diffraction (XRD) of iron oxide nanoparticles (IONPs) (**a**) and iron-silver bimetallic nanoparticles (IO@AgNPs) (**b**)

The crystalline nature of nanoparticles was confirmed by XRD analysis. Figure 2 shows the XRD pattern of IONP and IO@AgNP nanoparticles. The diffraction peaks of IONPs appearing at 30.1, 35.3, 42.9, 53.3, 56.8, and 62.3 correspond to 220, 311, 400, 422, 511, and 440 facets of the face-centered cubic crystal structure, respectively, indicating a pure iron nanoparticle preparation (Rasouli et al. 2018). While in the presence of silver, the diffraction peaks of IO@AgNPs appear at 37.9, 44.1, 64.2, 77.8, and 81.0 corresponding to 111, 200, 220, 311, and 222 respectively (Najafpoor et al. 2020). The average crystallite size of IONPs and IO@AgNPs according to Scherrer equation calculated using the width of the diffraction peaks is found to be 14 ± 5 and 46 ± 8 nm approximately in agreement with the particle size obtained from the TEM image (de Oliveira Gonçalves et al. 2020).

**Fig. 3:**
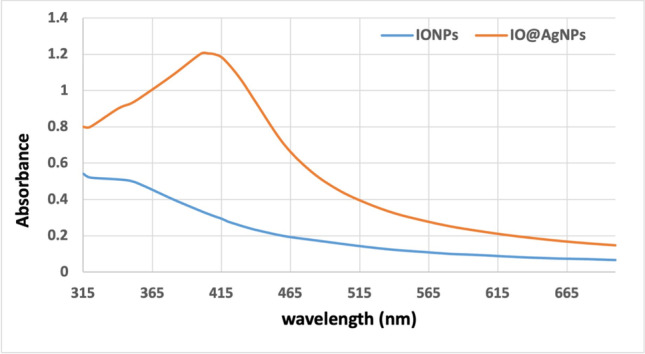
UV–visible spectra of iron oxide nanoparticles (IONPs) and iron-silver bimetallic nanoparticles (IO@AgNPs)

The absorption spectra were used to confirm the structure of nanoparticles. Figure 3 shows the characteristic surface plasmon resonance (SPR) band at 415 nm for IO@AgNPs indicating the formation of silver nanoparticles while iron nanoparticles had no significant peak in this region (Elbialy et al. 2014 and Sridharan et al. 2013).

**Fig. 4:**
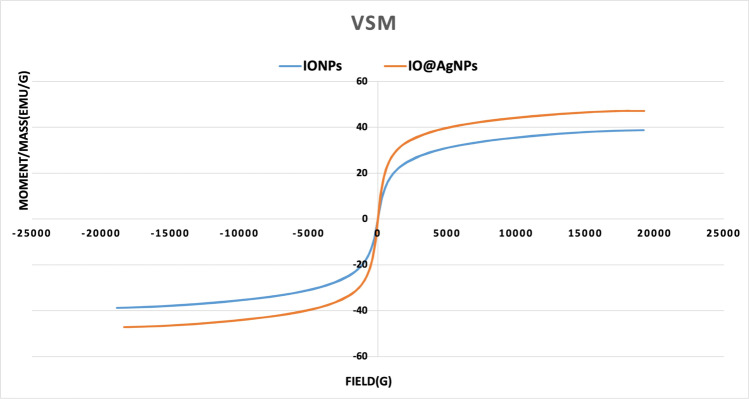
Vibrating sample magnetometer (VSM) of iron oxide nanoparticles (IONPs) and iron-silver bimetallic nanoparticles (IO@AgNPs)

The magnetic properties of the IONPs and IO@AgNPs are recorded using VSM (Fig. 4). The analysis showed that the saturation magnetization of IONPs was 38.7 emu/g while IO@AgNPs showed a higher value in saturation magnetization “47.2 emu/g”. The higher value occurred when silver was added to the surface of iron oxide and this result agrees with Najafpoor et al. (2020) and Zhao et al. (2019). Moreover, the superparamagnetic behavior was noted from the magnetization curve, where both the residual and coercivity forces were near to zero. Superparamagnetic nanoparticles permit a well control over the application of their magnetic properties because they require a strong response to an external magnetic field. As a result, IONPs and IO@AgNPs’ medical application uses are increased.

Delivering the maximal radiation to the target tumor tissue while saving the surrounding normal tissue is the aim of radiotherapy. In order to reduce the toxicity for normal tissues, patients are treated with fractionated doses over a period of several weeks. However, in advanced treatment planning, the maximum dose is deposited as a single dose, where the energy is sufficient to start the biochemical ROS production to destroy the proliferation of the malignant cells (Monem et al. 2020). Consequently, radiobiological effects are crucial to the effort to overcome the common problem with RT, such as the inability to distinguish between tumor and healthy cells due to a lack of tissue selectivity. The most modern strategies make use of NPs to maximize the localized therapeutic effect of RT, lower the local dose to be provided and the toxicity to normal surrounding tissue, and thereby improve the patient’s quality of life during and after radiological treatments (Moding et al. 2013 and Gong et al. 2021).

Cells produce a specific quantity of ROS under physiological circumstances, and the antioxidant system controls their concentration to preserve cellular equilibrium. One of the most effective ways to produce free radicals is using ionizing radiation. As a result, radiation-induced ROS generation disturbs the cellular balance and causes oxidative stress, which can result in DNA damage and other cell death mechanisms (Klein et al. 2014).

**Fig. 5:**
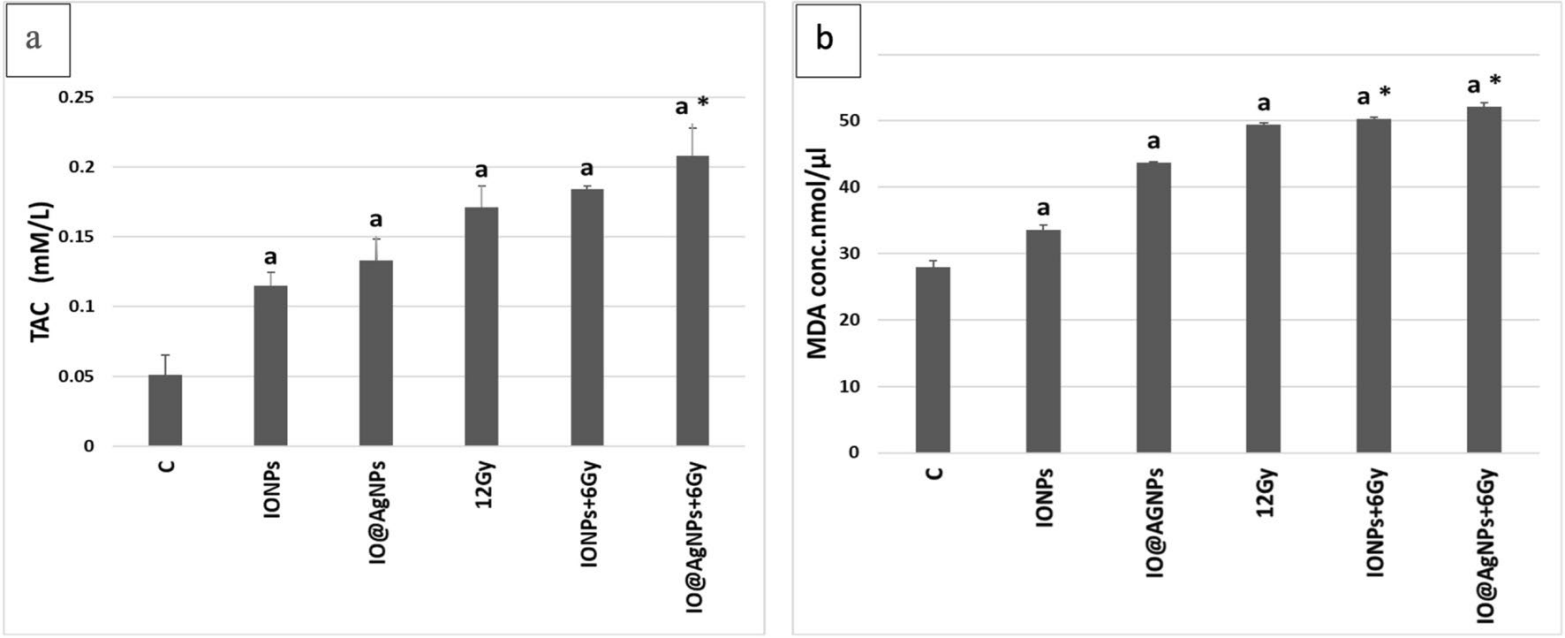
Total antioxidant capacity (TAC) (**a**) and malondialdehyde (MDA) (**b**) levels in tumor tissue for the different groups: The control mice group (C) and mice groups treated with iron oxide nanoparticles (0.8 mg/mL) (IONPs), iron-silver nanoparticles (1.6 mg/mL) (IO@AgNPs), high radiation dose (HRD) (12 Gy), iron oxide nanoparticles (0.8 mg/mL) and low radiation dose (LRD) (6 Gy) (IONPs + 6 Gy), and iron-silver nanoparticles (1.6 mg/mL) and LRD (IO@AgNPs + 6 Gy). The nanoparticles and radiation doses were fractionated equally into 3 sessions during the treatment protocol. The data points are represented as mean ± SD (*n* = 5) (a, *p* < 0.001) compared to the control group (C) and (*, *p* < 0.001) compared to HRD group (12 Gy)

TAC and MDA are considered biomarkers of oxidative stress, they are products of lipid peroxidation, and their levels are interrelated to tumor progression. Figure 5a shows a significant increase in TAC in all treated groups compared to the control. In addition, the IO@AgNPs + 6 Gy group showed a significant increase in TAC by about 21% compared to the 12 Gy-treated group.

However, the determination of only TAC gives insufficient information about the antioxidant situation, as it does not measure all antioxidant parameters. Consequently, MDA was used to test the effects of treatment with nanoparticles and radiation on lipid peroxidation. In Fig. 5b, there was a significant increase in IONPs, IO@AgNPs, 12 Gy, IONPs + 6 Gy, and IO@AgNPs + 6 Gy groups by about 20, 56, 76, 80, and 86% respectively compared to the control group and the percentage increases in IONPs + 6 Gy and IO@AgNPs + 6 Gy groups compared to 12 Gy group were nearly 2 and 6% respectively. These results indicate that radiation causes an increase in free radicals such as ROS that alter the antioxidant state of cells and generates apoptosis. Additionally, autophagy, a strong defense against oxidative stress (OS) damage, can kill cancer cells by inactivating autophagy-related genes and blocking the autophagy-negative regulator (Ferdous and Yusof 2021).

On the other hand, radiation therapy causes electron leakage from the electron transport chain, resulting in a rise in hydrogen peroxide levels. The transition from hydrogen peroxide to the extremely reactive hydroxyl radical can then be catalyzed by IONPs (Hauser et al. 2016). With excessive production of free radicals, oxidative stress will result, which causes damage to macromolecules such as lipids that could induce lipid peroxidation in vivo, leading to the degeneration of tissues (Gupta et al. 2004).

Our findings show that IO@AgNPs have a significant ability to improve radiosensitivity of solid Ehrlich carcinoma cells more than IONPs. The radiosensitivity improvement of IO@AgNPs is caused by a high decrease in cytoprotective autophagy, followed by an increase in calcium-dependent apoptosis (Zhang et al. 2018).

Moreover, nanoparticles have inherent toxicities and could be used to enhance cancer treatments. Paunovic et al. (2020) reported that the IONPs may not only improve the influence of gamma-rays on ROS generation but may also act as catalysts due to their surfaces by releasing iron ions that then react with oxygen and hydrogen peroxide in the cytoplasm via Fenton reaction to form ROS.

On the other hand, iron-silver bimetallic nanoparticles show a higher cytotoxicity performance than IONPs as the presence of silver nanoparticles increased the toxicity of iron oxide. The arrangement of ion chains in the cell membrane resulted in a high affinity for nanoparticles, which could inhibit transcriptional regulation and protein synthesis, causing cell death (de Oliveira Gonçalves et al. 2020). These results are consistent with the previous studies in Hauser et al. (2016), Klein et al. (2014), and Paunovic et al. (2020).

**Fig. 6:**
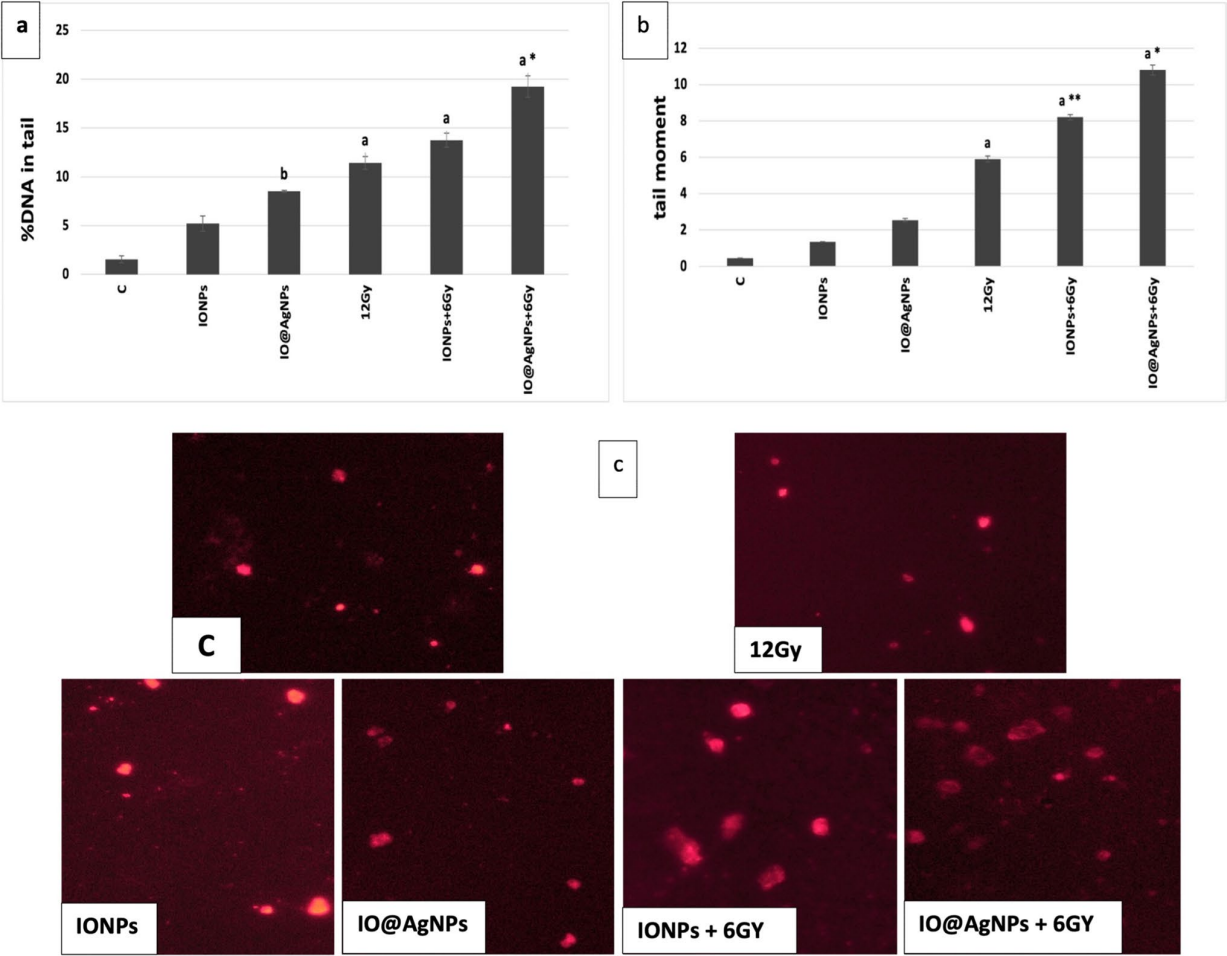
Comet parameters observed in %DNA in tail (**a**), tail moment (**b**), and comet images (**c**) of tumor in all experimental groups: The control mice group (C) and mice groups treated with iron oxide nanoparticles (0.8 mg/mL) (IONPs), iron-silver nanoparticles (1.6 mg/mL) (IO@AgNPs), high radiation dose (HRD) (12 Gy), iron oxide nanoparticles (0.8 mg/mL) and low radiation dose (LRD) (6 Gy) (IONPs + 6 Gy), and iron-silver nanoparticles (1.6 mg/mL) and LRD (IO@AgNPs + 6 Gy). The nanoparticles and radiation doses were fractionated equally into 3 sessions during the treatment protocol. The data points are represented as mean ± SD (*n* = 5) (a, *p* ≤ 0.0001; b, *p* ≤ 0.03) compared to the control group (C) and (*, *p* ≤ 0.001; **, *p* ≤ 0.008) compared to HRD group (12 Gy)

In the investigations of radiation-induced cell death, DNA is the only attractive objective. This is because if other cell structures and organelles are damaged, the DNA can replicate them (Zhang et al. 2022). In the present study, comet assay is well recognized as the combination treatment altered the induction of DNA damage in the Ehrlich tumor. The damage of DNA is reflected by the increase in the comet parameter (DNA (%) in tail and tail moment). The present study reported that the damage of DNA induced by LRD is enhanced by NPs, so the damage is radiosensitizing-dependent (Fig. 6). Figure 6 shows that the DNA (%) in tail significantly increased by about 68% in the IO@AgNP group compared to the HRD group. Figure 6b illustrates the tail moment increased by 39 and 83% in IONPs + 6 Gy and IO@AgNPs + 6 Gy groups respectively compared to HRD (12 Gy) group. These findings were corroborated by the comet images in Fig. 6c, which demonstrated that there was significant cell damage in both groups as seen by the production of comet tails (ghost cells). Based on the integrated results, the combination treatment (NPs and low radiation dose) has superior therapeutic efficacy against Ehrlich tumor over the high radiation dose. According to Russell et al.’s (2021) study, NPs enhanced the low dose of radiation by increasing the production of ROS, subsequently increasing the effect of radiation on the DNA compared to the high dose.

**Fig. 7:**
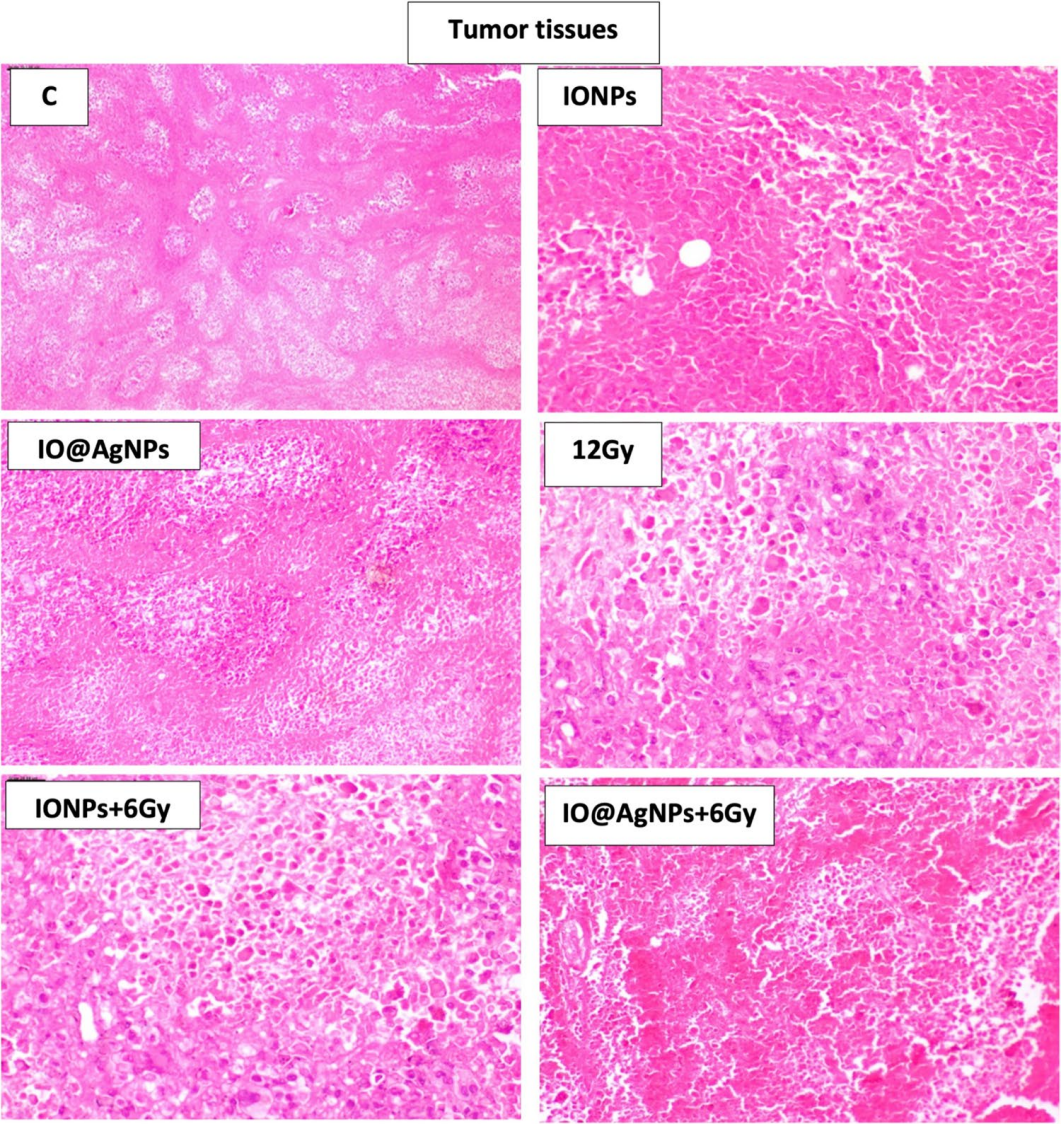
Tumor histopathology photomicrographs in control mice group (C) and mice groups treated with iron oxide nanoparticles (0.8 mg/mL) (IONPs), iron-silver nanoparticles (1.6 mg/mL) (IO@AgNPs), high radiation dose (HRD) (12 Gy), iron oxide nanoparticles (0.8 mg/mL) and low radiation dose (LRD) (6 Gy) (IONPs + 6 Gy), and iron-silver nanoparticles (1.6 mg/mL) and LRD (IO@AgNPs + 6 Gy). The nanoparticles and radiation doses were fractionated equally into 3 sessions during the treatment protocol

Increased cell death is a result of increased intracellular H_2_O_2_, and DNA damage caused by radiation and NP treatment (Askar et al. 2022), as demonstrated by histological analysis of the tissues from Ehrlich tumors in Fig. 7. Histopathological examination of tumor sections of untreated mice showed a typical picture of Ehrlich carcinoma infiltrating thigh muscles. Treated tumors with IONPs and IO@AgNPs showed less severe invasion of carcinoma cells. Tumor treatment with HRD induced variable degrees of necrosis and anaplasia in cancer cells. However, LRD combined with IONPs showed increased areas of necrotic and apoptotic cells while IO@AgNP groups showed more regression of tumor invasion and massive areas of necrosis (Sayed et al. 2021and Rageh and El-Gebaly 2019).

**Fig. 8:**
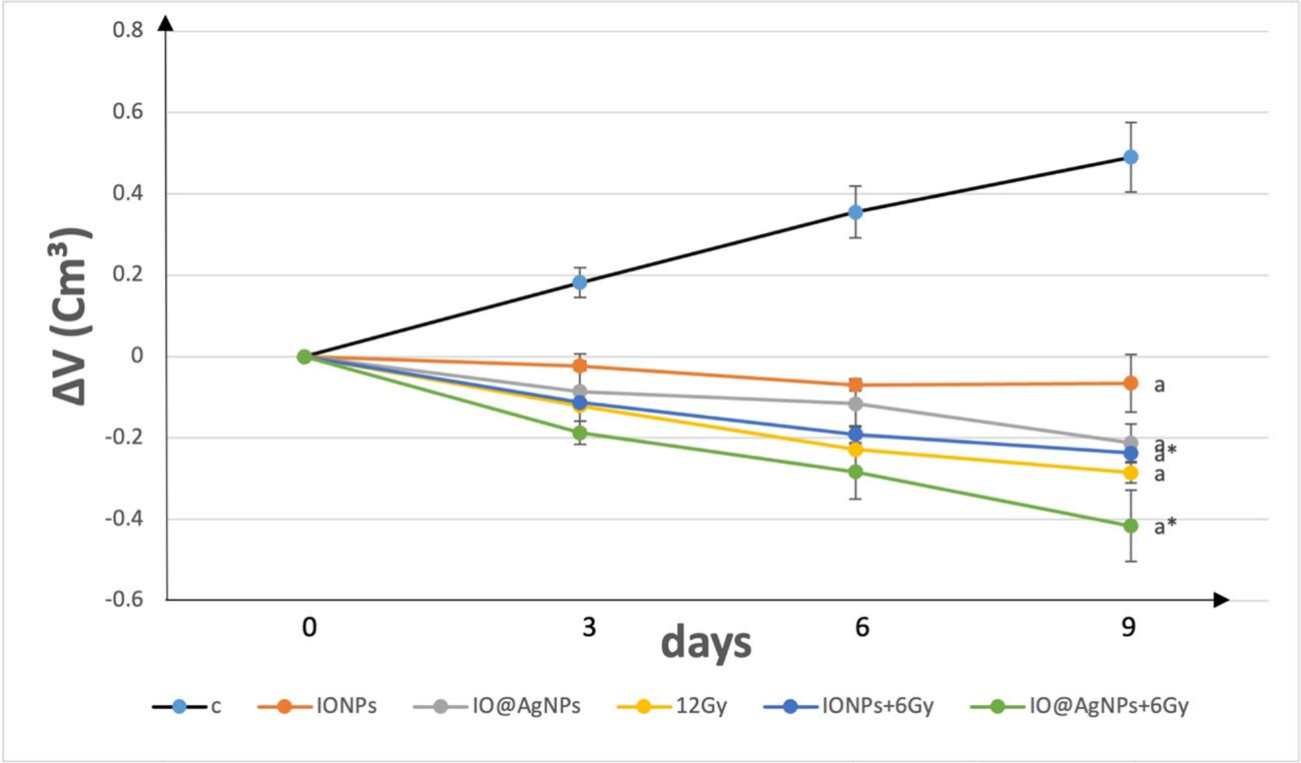
Average change in the tumor size (cm.^3^) in control mice group (C) and mice groups treated with iron oxide nanoparticles (0.8 mg/mL) (IONPs), iron-silver nanoparticles (1.6 mg/mL) (IO@AgNPs), high radiation dose (HRD) (12 Gy), iron oxide nanoparticles (0.8 mg/mL) and low radiation dose (LRD) (6 Gy) (IONPs + 6 Gy), and iron-silver nanoparticles (1.6 mg/mL) and LRD (IO@AgNPs + 6 Gy). The nanoparticles and radiation doses were fractionated equally into 3 sessions during the treatment protocol. The data points are represented as mean ± SD (*n* = 5) (a, *p* ≤ 0.001) compared to the control group (C) and (*, *p* < 0.03) compared to HRD group (12 Gy)

The effectiveness of the suggested treatments (NPs and low radiation dose) was assessed by tracking the tumor’s growth over the period of treatment. The solid Ehrlich tumor tissues showed an obvious decrease in size and a delay in their growth. Figure 8 and Table 1 show a significant decrease in tumor volume in all treated groups at the end of treatment protocol (day 9) compared to the control group by about 1.1, 1.4, 1.5, 1.5, and 1.8-fold for IONPs, IO@AgNPs, 12 Gy, IONPs + 6 Gy, and IO@AgNPs + 6 Gy respectively. The above results indicate that the combination thereby (bimetallic NPs and LRD) is more effective than HRD (Askar et al. 2022).

**Table 1:** Average change in the tumor size (cm^3^) in control mice group (C) and mice groups treated with iron oxide nanoparticles (0.8 mg/mL) (IONPs), iron-silver nanoparticles (1.6 mg/mL) (IO@AgNPs), high radiation dose (HRD) (12 Gy), iron oxide nanoparticles (0.8 mg/mL) and low radiation dose (LRD) (6 Gy) (IONPs + 6 Gy), and iron-silver nanoparticles (1.6 mg/mL) and LRD (IO@AgNPs + 6 Gy). The nanoparticles and radiation doses were fractionated equally into 3 sessions during the treatment protocol. The data points are represented as mean ± SD (*n* = 5) (a, *p* ≤ 0.001) compared to the control group (C) and (*, *p* < 0.03) compared to HRD group (12 Gy)

Average change in tumor size (cm^3^)				
Days				
Groups	0	3	6	9
C	0	0.18 ± 0.03	0.35 ± 0.06	0.49 ± 0.08
IONPs	0	− 0.02 ± 0.01	− 0.06 ± 0.01	− 0.06 ± 0.07^a^
IO@AgNPs	0	− 0.08 ± 0.09	− 0.11 ± 0.05	− 0.21 ± 0.04^a^
12 Gy	0	− 0.12 ± 0.01	− 0.22 ± 0.01	− 0.28 ± 0.03^a^
IONPs + 6 Gy	0	− 0.11 ± 0.01	− 0.19 ± 0.02	− 0.23 ± 0.01^a*^
IO@AgNPs + 6 Gy	0	− 0.18 ± 0.03	− 0.28 ± 0.06	− 0.41 ± 0.08^a*^

**Fig. 9:**
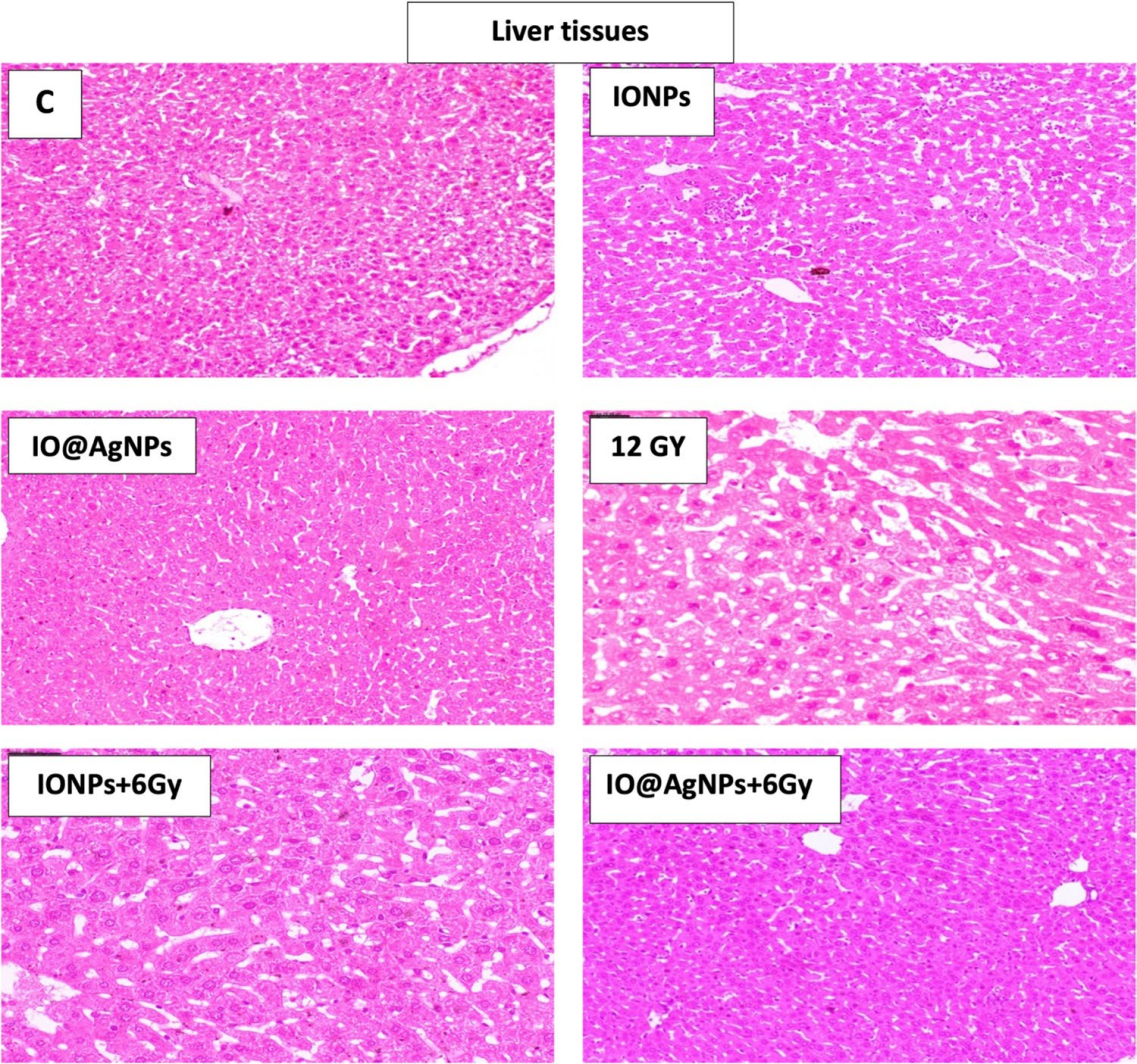
Liver histopathology photomicrographs in control mice group and mice groups treated with iron oxide nanoparticles (0.8 mg/mL) (IONPs), iron-silver nanoparticles (1.6 mg/mL) (IO@AgNPs), high radiation dose (HRD) (12 Gy), iron oxide nanoparticles (0.8 mg/mL) and low radiation dose (LRD) (6 Gy) (IONPs + 6 Gy), and iron-silver nanoparticles (1.6 mg/mL) and LRD (IO@AgNPs + 6 Gy). The nanoparticles and radiation doses were fractionated equally into 3 sessions during the treatment protocol

The biosafety issue must be taken into consideration before recommending any suggested therapeutic approach for preclinical trials. The cytotoxicity of the suggested method must be assessed in the liver because it is the first organ where foreign toxins accumulate (Rageh et al. 2018). The amount of amelioration (ALT) and histopathological change were used to assess the oxidative stress in liver tissues. When the liver gets damaged or inflamed, it can release ALT into the bloodstream. This causes the ALT levels to rise. Table 2 shows a significant increase in all treated groups compared to the control group indicating the presence of liver inflammation with different ratios. The small size and the active surface of nanoparticles especially the iron nano coated by silver are behind this elevation, which can penetrate and damage the liver cell membrane and release its enzymes into the bloodstream. But the inflammation that occurred in the HRD group shows a higher value when compared to IO@AgNPs + 6 Gy groups by about 47%. So far, the high radiation dose harmed cell membrane integrity, causing the liver to release its enzymes, resulting in a rise in serum ALT and this agrees with the study in Mekkawy et al. (2020). Moreover, the degree of liver inflammation that occurred due to different treatments by nanoparticles and ionizing radiation could be observed in the histopathology examination of liver tissues (Fig. 9). The control group “untreated mice” showed high destruction of hepatic tissues and infiltration of the liver with the tumor cells. Treated mice with IONPs alone showed almost similar findings. However, the IO@AgNP group showed less severe inflammation and degeneration of hepatocytes.

**Table 2:** Alanine transaminase (ALT) level in serum of control group that contain EST and received no treatment and treated groups IONPs, IO@AgNPs, HRD (12 Gy), IONPs + 6 Gy, and IONPs + 6 Gy. The data points are represented as mean ± SD (*n* = 5) (a, *p* ≤ 0.001) compared to the control group and (*, *p* < 0.001) compared to HRD group (12 Gy)

Groups	ALT level in serum (IU/L)
Control	68 ± 2
IONPs	96 ± 3^a^
IO@AgNPs	98 ± 2^a^
12GY	116 ± 5^a^
IONPs + 6 Gy	88 ± 3^a*^
IO@AgNPs + 6 Gy	61 ± 4^a*^

Regarding experimental groups that receive radiation therapy, the HRD group induced severe necrosis and damage to liver tissues (Kim and Jung 2017). On the other hand, histological sections of groups treated with LRD combined with IONPs showed moderate affection for the liver while the IO@AgNP group showed almost normal liver tissues with minimal destruction and infiltration with inflammatory cells.

Based on the previously mentioned results, even though the mice treated with bimetallic nanoparticles (IO@AgNPs) and LRD showed a negligible difference in oxidative stress compared to the mice treated with IO@NPs + 6 Gy, this group showed a significant difference in DNA damage and tumor size by about 30 and 40% respectively compared to IONPs, which indicates a benefit of using iron and silver in one hybrid nanoparticle for more enhancement to the LRD and consequently enhance cancer treatment.

However, more investigation is still needed to decrease nanoparticle toxicity by modifying their surface with a biocompatible shell that is relatively nontoxic to normal tissues.

## Conclusion

A quick and secure approach was used to create IONPs and IO@AgNPs, and they were then characterized. Solid Ehrlich tumors injected in Balb/c mice were successfully treated with low radiation doses using NPs, which have been shown to be efficient radiosensitizers. According to our integrated data, combination therapy (NPs + radiation) demonstrated great therapeutic efficacy against Ehrlich tumor with negligible side effects over high radiation dose.

## Data Availability

Not applicable.
